# Antimicrobial efficacy of light-cured glass ionomer cements incorporating red propolis extract

**DOI:** 10.2340/biid.v12.44895

**Published:** 2025-10-29

**Authors:** Maria Helena Nunes Borges, Thays Maria de Oliveira Almeida, Pedro Henrique Sette-de-Souza, Mayara Abreu Pinheiro, Basílio Rodrigues Vieira, Gêisa Aiane de Morais Sampaio

**Affiliations:** Department of Dentistry, University of Pernambuco, Arcoverde, Pernambuco, Brazil

**Keywords:** Glass ionomer cements, propolis, antimicrobial action

## Abstract

**Objective:**

The aim of the present study was to evaluate the antimicrobial activity of resin-modified glass ionomer cements (RMGICs) supplemented with red propolis ethanolic extract (RPEE) against *Streptococcus mutans* cultures.

**Materials and methods:**

Four RMGICs (Gold Label, Ionolux, Vitro Fill, and Riva) were used with the addition of RPEE at concentrations of 11% and 20%. For the control groups, the cements were manipulated and light-cured according to the manufacturers’ instructions. *S. mutans* strains from stock cultures were grown in brain heart infusion (BHI) broth. Cement samples were placed in direct contact with the microorganism for 48 h in a bacteriological incubator. After this period, the diameters of the inhibition zones were measured using a digital caliper.

**Results:**

Data analysis showed a significant increase in the size of the inhibition zones for Gold Label and Vitro Fill RMGICs in the groups with 11% RPEE, compared to their respective controls. When comparing different cement brands containing 11% RPEE, Gold Label and Vitro Fill exhibited significantly larger inhibition zones than Ionolux.

**Conclusion:**

The addition of 11% RPEE was shown to enhance the antimicrobial activity of two of the four tested RMGICs, with Gold Label and Vitro Fill demonstrating the greatest antimicrobial potential following supplementation with RPEE.

## Introduction

Glass ionomer cements (GICs) are widely used in dentistry due to their favourable properties, including fluoride release and adhesion to tooth structure [1–3]. They are classified into conventional and resin-modified types, with the latter combining advantages of both composite resins and traditional glass ionomers [4]. Resin-modified glass ionomer cements (RMGICs) exhibit improved esthetics, moisture resistance, and toughness compared to conventional GICs. Both types undergo an acid-base reaction, but RMGICs also set through polymerization [4]. Glass ionomer cements have various applications in dentistry, including as liners, bases, luting agents, and restorative materials. While GICs offer numerous benefits, they still face challenges in terms of long-term wear and strength. Ongoing research aims to enhance their properties without compromising their inherent qualities of adhesion and fluoride release [5, 6].

The cariostatic properties of GICs are considered to be primarily due to the incorporation of fluoride released by the cement into the dental structure, thereby making it more resistant to bacterial acid attack. However, this alone is not sufficient to completely inhibit bacterial growth [2, 3]. Nevertheless, studies have shown that the addition of antimicrobial agents to GICs can significantly enhance the antimicrobial properties of these materials [3, 7–9].

Propolis is a natural substance produced by bees from resins collected from plant stems. Interest in its therapeutic use has grown considerably due to scientific evidence supporting its role in disease prevention, attributed to its various biological properties, including antimicrobial, anti-inflammatory, antioxidant, antiviral, and wound-healing activities [10–14].

Red propolis is found in the northern and northeastern regions of Brazil, especially in coastal areas. Although still underexplored, it has demonstrated promising antimicrobial and antifungal activity [15]. Due to its antibacterial action against oral microorganisms such as *Streptococcus mutans*, the incorporation of Red Propolis Ethanolic Extract (RPEE) into GICs may enhance their anticariogenic potential. The use of this modified material could contribute to the reduction of bacterial counts in the area adjacent to the restoration [9, 16]. The aim of the present study was, therefore, to evaluate the antimicrobial activity, against *S. mutans* cultures, of RMGICs supplemented with RPEE. The alternative hypothesis is that the addition of RPEE enhances the antimicrobial effect of the tested RMGICs.

## Materials and methods

The analyses were conducted at the Biotechnology Laboratory of the University of Pernambuco.

### Preparation of glass ionomer cements containing red propolis ethanolic extract

The present study used RPEE at concentrations of 11% and 20%, sourced from the coastal region of Alagoas, Brazil (Fernão Velho, Alagoas, Brazil, Batch 03/23). Four restorative RMGICs were used: Riva Light Cure (SDI, Victoria, Australia), Ionolux (VOCO, Cuxhaven, Germany), Vitro Fill (DFL, Lyon, France), and Gold Label (CG Corporation, Tokyo, Japan) (Table 1). For the control groups, the cements were manipulated according to the manufacturers’ instructions. In the experimental groups, RPEE solutions at concentrations of 11% and 20% were incorporated into the cement liquid during manipulation, using a 1:1 ratio (one drop of tartaric acid-based liquid to one drop of RPEE solution), measured with the same dispensing tip, and subsequently spatulated with the powder.

**Table 1:** Manufacturer, composition, and batch of the RMGICs tested.

GIC	Manufacturer	Liquid	Powder	Batch
Riva Light Cure	SDI (Brazil)	Polyacrylic acid mixture, distilled water, tartaric acid, 2-hydroxyethyl methacrylate (HEMA), dimethacrylate acidified monomer	Fluoro-alumino-silicate glass	120949A
Ionolux	VOCO (Germany)	Polyacrylic acid, HEMA, water, UDMA, GlyDMA, initiators, stabilisers, pigments	Fluorosilicate glass and strontium	2325500381
Vitro Fill LC	DFL (France)	Polyacrylic acid, Tartaric acid, benzoyl peroxide, HEMA, camphorquinone and distilled water.	Fluorine strontium aluminium silicate, dehydrated polyacrylic acid and iron oxide	11875203
Gold Label 2 Light Cure	GC corporation (Japan)	Polyacrylic acid, water, and tartaric acid	Strontium-based Fluoro-aluminosilicate glass	2305291

GIC: glass ionomer cement.

### Antimicrobial analysis

The materials were placed in polyethylene moulds (7 mm × 3 mm), with the surface covered by a glass slide, and allowed to set for 5 min at 25°C. A total of 120 samples (*n* = 10) were fabricated for each test and distributed across 12 groups: Group RC (Riva Light Cure Control), Group R11 (Riva Light Cure with 11% RPEE), Group R20 (Riva Light Cure with 20% RPEE), Group IC (Ionolux Control), Group I11 (Ionolux with 11% RPEE), Group I20 (Ionolux with 20% RPEE), Group VC (Vitro Fill Control), Group V11 (Vitro Fill with 11% RPEE), Group V20 (Vitro Fill with 20% RPEE), Group GC (Gold Label 2 Light Cure Control), Group G11 (Gold Label 2 Light Cure with 11% RPEE), and Group G20 (Gold Label 2 Light Cure with 20% RPEE).

*S. mutans* ATCC 25175 bacterial strains were cultured in Brain Heart Infusion (BHI). A 10^–^¹ dilution containing 1.2 × 10⁸ CFU/mL was prepared, determined through serial dilution in 0.85% saline solution. After incubation at 37°C for 48 h, the bacterial strain was spread on BHI agar plates and left at room temperature for 30 min. Subsequently, the samples were placed in direct contact with the medium and incubated at 37°C for 48 h in a microaerophilic environment. After this period, the diameters of the inhibition zones were measured at two points, horizontally and vertically, using a digital caliper.

### Statistical analysis

The results were organised into a database using Microsoft Excel and then exported to the Statistical Package for the Social Sciences (SPSS, version 20, SPSS Inc., Chicago, IL, USA) for statistical analysis. The non-parametric Kruskal-Wallis test, followed by Dunn’s multiple comparison test, was used to compare antimicrobial effect results. The tests were conducted with a significance level of 95% (*p* < 0.05).

## Results

**Figure 1:**
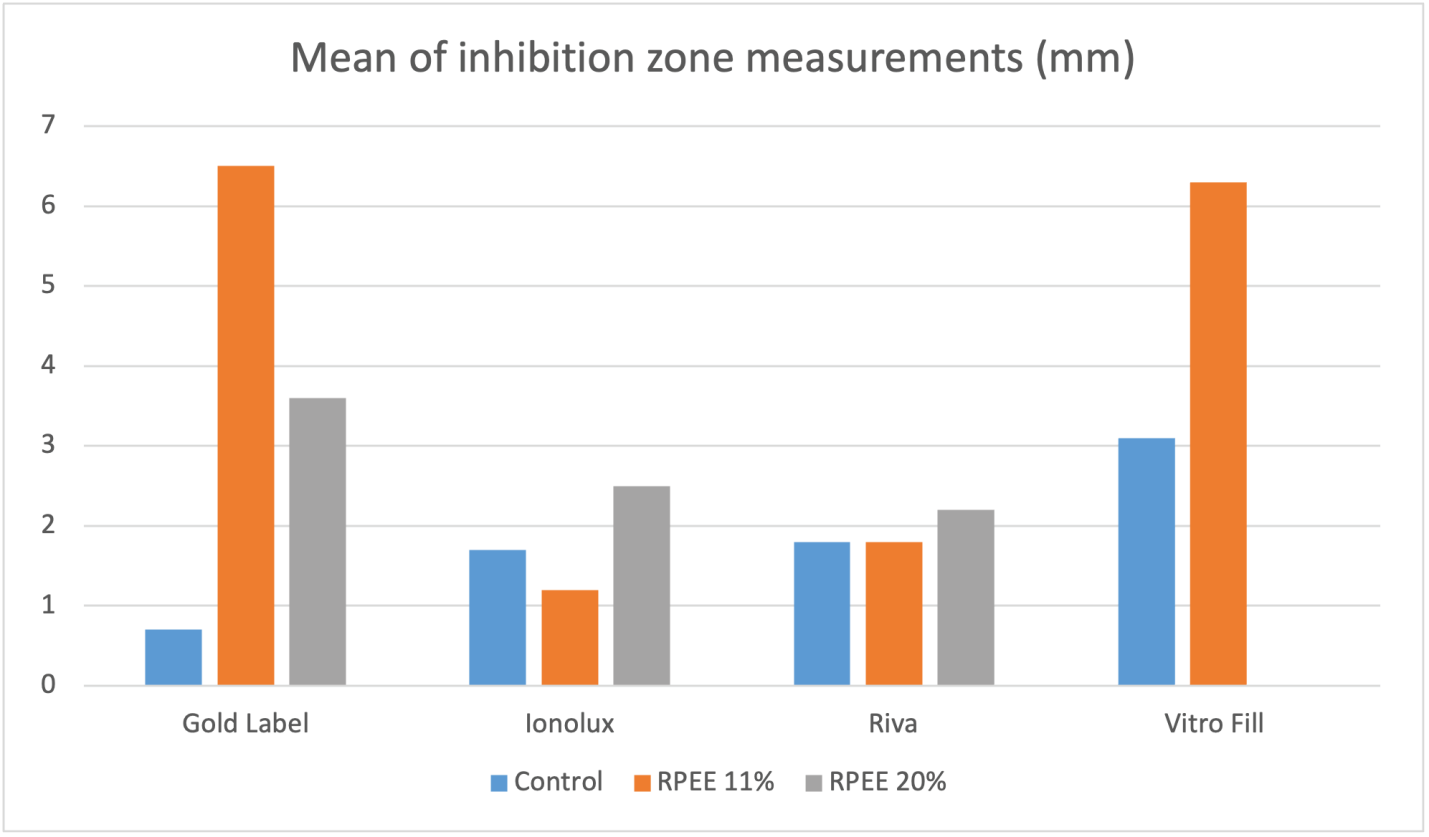
Mean of inhibition zone measurements.

For the Gold Label and Vitro Fill LC RMGICs, data analysis revealed an increase in the diameter of the inhibition zones in the groups with the addition of 11% RPEE, with statistically significant differences compared to their respective control groups (*p* = 0.001 and *p* = 0.009, respectively) (Figure 1). In the case of Ionolux and Riva cements, an increase in inhibition zone size was also observed in the groups with the addition of red propolis; however, these differences were not statistically significant compared to their controls (Table 2).

**Table 2:** Mean and standard deviation of inhibition zone measurements (mm).

	Gold label	Ionolux	Riva	Vitro fill	p*
Control	0.7 (0.1)^A,a^	1.7 (0.3)^AB,ab^	1.8 (0.5)^ab^	3.1 (0.7)^A,b^	**0.002**
**RPEE 11%**	6.5 (1.0)^B,a^	1.2 (0.4)^A,b^	1.8 (0.8)^ab^	6.3 (1.3)^B,a^	**0.002**
**RPEE 20%**	3.6 (1.0)^AB^	2.5 (0.5)^B^	2.2 (1.1)	-	0.052
p*	**0.001**	**0.007**	0.697	**0.009**	-

RPEE: red propolis ethanolic extract.

Means followed by the same letters do not express a statistically significant difference (*p* > 0.05). Means followed by different letters express a statistically significant difference (*p* < 0.05).

A,a Capital letters express differences between the control and test groups in the same cement (in a column); lowercase letters express the comparison between the cements (in a row).

* Non-parametric Kruskal-Wallis test, followed by Dunn’s multiple comparisons test.

When comparing the different concentrations of RPEE tested, it was found that, for Ionolux and Riva, the inhibition zones of the cements containing 20% RPEE were larger than those with 11% RPEE, with a statistically significant difference observed only between groups I11 and I20 (*p* = 0.007). Conversely, Gold Label cement showed a reduction in the size of the inhibition zones in the 20% RPEE group compared to the 11% group, although without statistical significance. The data from the Vitro Fill LC group with 20% RPEE were not analysed, as the addition of propolis appeared to interfere with the setting of the cement (Table 2).

In the comparison of results among the different brands of cements tested, Vitro Fill LC demonstrated the greatest antimicrobial activity, with a statistically significant difference compared to Gold Label (*p* = 0.002), when considering only the control groups. Among the cements with 11% RPEE, Gold Label and Vitro Fill LC presented significantly larger inhibition zones than Ionolux (*p* = 0.002) (Figure 1). For the groups containing 20% RPEE, no statistically significant differences were found among the cements tested (Table 2).

## Discussion

Studies have investigated the antimicrobial effects of GIC supplemented with propolis against *S. mutans*, and this natural product has demonstrated significant antibacterial properties when added to GICs [3, 17]. The addition of propolis to RMGICs at concentrations of 15% and 25% also showed increased inhibitory effects on *S. mutans* growth in Saputra et al.’s study [18], though these results were not observed in Panahandeh et al.’s study [19], likely due to the use of water as a solvent instead of ethanol. Udoye et al. [20] demonstrated that ethanol extract of propolis affects a broader spectrum of bacteria and exhibits higher bactericidal effects compared to aqueous extract.

In the present study, the antimicrobial action against *S. mutans* of RMGICs with the addition of RPEE at concentrations of 11% and 20% was evaluated. These concentrations were selected based on previous studies that tested propolis incorporation into dental materials and demonstrated antimicrobial efficacy without compromising handling properties of the cements [3, 18]. Pilot tests performed in our laboratory also indicated that concentrations higher than 20% impaired the setting reaction and consistency of some RMGICs. Therefore, we chose these intermediate concentrations to ensure a balance between antibacterial efficacy and adequate cement manipulation.

*S. mutans* was chosen because it is one of the primary aetiological agents of dental caries, strongly associated with biofilm formation, acidogenicity, and demineralisation processes. Previous studies on GIC modifications have also used *S. mutans* as the reference strain [7, 9], allowing comparison of our findings with the literature. The agar diffusion test (direct contact) was chosen as it is a simple, reproducible, and widely used method to screen antibacterial activity of restorative materials. Its merit lies in allowing the detection of diffusion-dependent antibacterial effects. However, the agar diffusion test limitation is that it does not fully replicate oral clinical conditions, such as biofilm complexity and salivary interactions.

The 48-h incubation period was chosen because it corresponds to the exponential growth phase of *S. mutans*, allowing clearer detection of antimicrobial effects. Moreover, this timeframe has been used in previous studies evaluating antibacterial activity of GICs [18, 21]. Clinically, this period is relevant as bacterial colonisation and biofilm formation around new restorations occur rapidly within the first 48 h.

The present study observed that two of the four RMGICs showed a larger inhibition zone against *S. mutans* following incorporation of 11% RPEE. These results are similar to Morais Sampaio et al.’s study [9], which found increased antimicrobial effects against both *S. mutans* and *Candida albicans* cultures using conventional GICs with RPEE concentrations of 25% and 50%. The enhanced antimicrobial efficacy of these materials can be attributed to the synergy between the bioactive components of red propolis (rich in flavonoids) and the intrinsic properties of GICs, such as fluoride release and low initial acidity. The interaction of these factors may facilitate the diffusion of antimicrobial compounds in the culture medium and enhance action against *S. mutans*.

The present study also evidenced that lower concentrations of RPEE can also cause significant increases in the size of inhibition zones of RMGICs in *S. mutans* cultures. For most cements tested, the antimicrobial action of cements with 11% RPEE was greater than that of cements with 20% RPEE, with the exception of Ionolux cement. This may be explained by the fact that higher concentrations of propolis increase resin matrix viscosity, which can reduce the diffusion of active compounds into the medium, thus limiting their antibacterial effect. Additionally, chemical interactions between flavonoids in propolis and the polymeric components of RMGICs may reduce the release of active agents at higher concentrations. However, these results differ from other studies that observed a concentration-dependent antimicrobial action of conventional GICs with addition of yellow PEE at concentrations of 10%, 25%, and 50% [3]; and RMGICs with addition of yellow propolis at concentrations of 15% and 25% [18].

The addition of red propolis to orthodontic adhesives has also shown to enhance their antimicrobial properties against *S. mutans* without altering their physical-mechanical properties of conversion degree and shear bond strength [22]. Similar results were observed in a study where conventional GICs containing 25% RPEE exhibited significantly increased antimicrobial activity against *S. mutans* and *C. albicans*, without affecting the mechanical properties and fluoride release of the cements [9]. However, studies have reported that high propolis concentrations may alter physical and mechanical properties such as compressive strength, setting reaction, and solubility [3, 19]. In our study, we observed handling difficulties with Vitro Fill at 20%, suggesting a potential compromise in cement properties. We suggested further mechanical property analysis as future research.

Previous investigations have demonstrated that red propolis exhibits favourable biocompatibility, promoting cell viability, and showing anti-inflammatory properties when incorporated into restorative materials [16, 23]. Sampaio et al. [16] demonstrated improved biocompatibility and tissue reaction when RPEE was added to restorative materials. Studies also evidenced the good antimicrobial action of red propolis against endodontic pathogens and promotion of cell viability of human dental pulp fibroblasts [23], indicating that adding RPEE to ionomeric materials can promote not only good antimicrobial action but also favourable biological responses from associated tissues. Therefore, when released from RMGICs, propolis is expected to have positive interactions with oral tissues.

In the present study, all tested RMGICs showed antimicrobial activity against *S. mutans*, with Vitro Fill LC cement standing out. These results demonstrate that RMGICs have good antimicrobial activity and corroborate with findings from other studies that RMGICs have greater antimicrobial activity against *S. mutans* than conventional GICs [24, 25]. Similarly, the study conducted by Fúcio et al. [21] found that RMGIC Vitremer was more effective in inhibiting *S. mutans* and allowed for greater pH neutralisation in the first 48 h than conventional GICs.

The differences observed between the cements can be attributed to variations in the composition of the RMGICs. For instance, Vitro Fill contains iron oxide and a distinct glass composition, which may enhance diffusion of antibacterial agents. Gold Label is strontium-based, which has been reported to contribute to antibacterial properties. Ionolux and Riva, on the other hand, contain additional resin monomers such as Urethane dimethacrylate (UDMA) and Glycidyl dimethacrylate (GlyDMA), which may hinder the release of bioactive compounds. These compositional aspects likely influenced the variability in inhibition zones.

Finally, the findings of the present study suggest that adding RPEE to restorative materials presents itself as a promising strategy, especially in clinical contexts with high caries risk, such as patients with high cariogenic activity or special needs, potentially reducing the risk of secondary caries and improving oral health outcomes. However, the present study focused only on the antibacterial effects, and it is also essential that future studies investigate the impact of the addition of RPEE on the mechanical properties, adhesion, chemical stability, and biocompatibility of RMGC, in addition to conducting clinical trials, to validate its safe and effective clinical use. Furthermore, quantifying the release profile of propolis compounds would provide important insights into their mechanism of action. We also suggest this as a direction for future studies.

## Conclusions

The addition of 11% RPEE was shown to enhance the antimicrobial activity of two of the four tested RMGICs, with Gold Label and Vitro Fill demonstrating the greatest antimicrobial potential following supplementation with RPEE.
